# 2. Space Time Trends of Community Onset *Staphylococcus aureus* Infections in Children Living in Southeastern United States: 2002-2016

**DOI:** 10.1093/ofid/ofab466.002

**Published:** 2021-12-04

**Authors:** Lilly Immergluck, Ruijin geng, Chaohua Li, Mike Edelson, Lance Waller, George Rust, Junjun Xu, Fatima Ali, Peter Baltrus, Traci Leong

**Affiliations:** 1 Morehouse School of Medicine, Atlanta, GA 30310, Georgia; 2 InterDev Inc., Roswell, Georgia; 3 Emory University, Atlanta, Georgia; 4 FLORIDA STATE UNIVERSITY, Atlanta, Georgia; 5 Ningbo Consulting Inc, Dunwoody, Georgia

## Abstract

**Background:**

Staphylococcus aureus (*S. aureus*) remains a serious cause of infections in the United States and worldwide. Methicillin susceptible *S. aureus* (MSSA) is the cause of half of all health care–associated staphylococcal infections, and Methicillin Resistant *S. aureus* (MRSA) is the leading cause of community onset skin and soft tissue infections in the US. This study looks at a 15-year trend of community onset (CO)-MRSA and MSSA infections and determines ‘best’ to ‘worst’ infection trends. We identified distinct groups of CO-MRSA and MSSA infection rate trajectories by grouping census tracts of the 20 county Atlanta Metropolitan Statistical Area (MSA) between 2002 to 2016 with similar temporal trajectories.

**Methods:**

This is a retrospective study from 2002-2016, using electronic health records of children living in Atlanta, Georgia with *S. aureus* infections and relevant US census data (at the census tract level). A group based trajectory model was applied to generate community onset *S. aureus* trajectory infection groups (low, high, very high) by census tract and were mapped using ArcGIS.

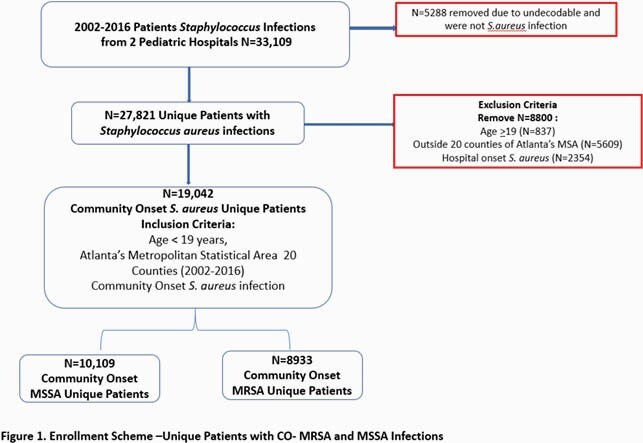

**Results:**

Three CO-MSSA infection groups (low, high, very high) and two CO-MRSA infection groups (low, high) were detected among 909 census tracts in the 20 counties. We found ~74% of all the census tracts with *S.aureus* occurrence during this time period belonged to low infection rate groups for both MRSA and MSSA, with a higher proportion occurring in the less densely populated counties. Census tracts in DeKalb County, one of Atlanta’s most densely populated areas, had the highest proportion of the worst infection trend patterns (CO-MRSA high or very high, CO-MSSA high or very high).

Trends of Community-Onset MRSA and MSSA Infection Rates Based on Group-based Trajectory Models

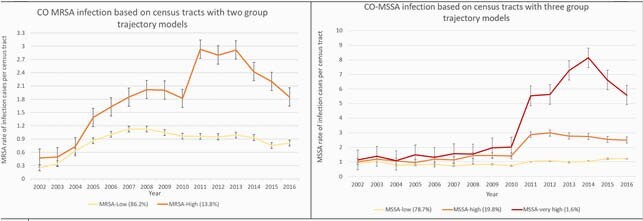

Spatial patterns for CO-MRSA and CO-MSSA Trajectory Trends in the Atlanta Metropolitan Area Between 2002 to 2016

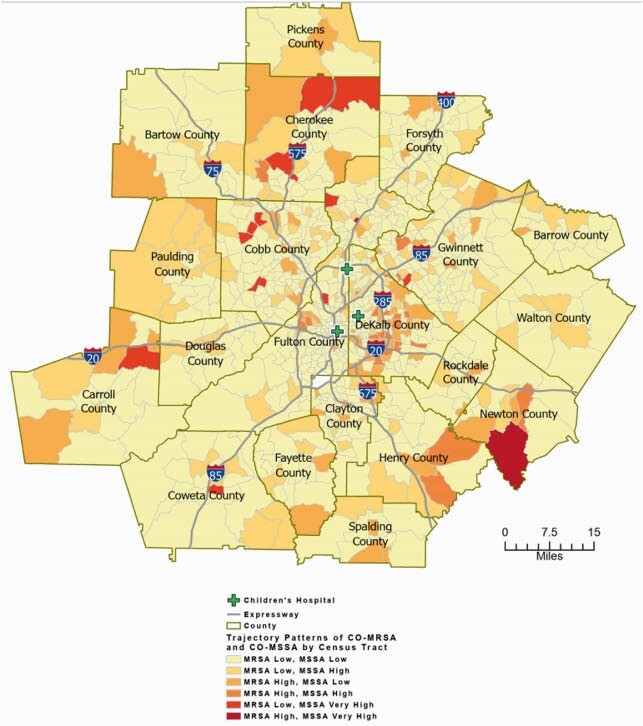

**Conclusion:**

Trends of *S. aureus* infection patterns, stratified by antibiotic resistance over geographic areas and time, identify communities with higher risks for MRSA infection compared to MSSA infection. Further investigation of the determinants of the trajectory groupings and the geographic outliers identified by this study may be a way to target prevention strategies aimed to prevent *S. aureus* infections.

**Disclosures:**

**All Authors**: No reported disclosures

